# Comparative evaluation of marginal adaptation and fatigue resistance of endodontically treated premolars restored with direct and indirect coronal restorations: an in vitro study

**DOI:** 10.1186/s12903-024-04382-8

**Published:** 2024-06-15

**Authors:** Shaimaa Mohammed Rohym, Heba Badra, Hanaa Nassar

**Affiliations:** 1 Conservative Dentistry Department, Faculty of Oral and Dental Medicine, Ahram Canadian University, 6th of October City, Egypt; 2 Endodontic Department, Faculty of Oral and Dental Medicine, Ahram Canadian University, 6th of October City, Egypt; 3 Fixed Prosthodontics Department, Faculty of Oral and Dental Medicine, Ahram Canadian University, 6th of October City, Egypt

**Keywords:** Margin adaptation, Ever-X posterior, Ribbond, Ceramic overlay, Fatigue resistance

## Abstract

**Background:**

An optimum restoration for reconstructing endodontically treated teeth should provide excellent marginal adaptation, high fracture resistance as well as maximum tooth structure conservation. The purpose of this study was to evaluate the marginal adaptation and fatigue resistance of different coronal restorations in endodontically treated premolars.

**Methods:**

Thirty sound maxillary first premolars were endodontically treated and received MOD cavities. Teeth were randomly allocated into three groups (*n* = 10) according to the type of coronal restoration: Group R: polyethylene fibers (ribbond), fibers-reinforced composite (everX posterior) and final layer of nano-hybrid composite. Group O: indirect lithium disilicate overlay and Group C: fiber-post, resin composite restoration, and lithium disilicate crown. Marginal gap assessment was performed before and after thermocycling (5000 cycles) using stereomicroscope. Samples were subjected to stepwise-stress loading starting at 200 N, and increased by 100 N in each step until failure occurred. Statistical analysis was done by One-way ANOVA followed Tukey`s Post Hoc test for multiple comparison. Paired t test was used to compare the marginal adaptation before and after thermocycling. Survival probability was evaluated by Life table survival analysis. Failure mode analysis was performed with Chi-square test.

**Results:**

Marginal gap was significantly the lowest in group R (37.49 ± 5.05) and (42.68 ± 2.38), while being the highest in group C (59.78 ± 5.67) and (71.52 ± 5.18) in before and after thermocycling respectively (*P* < 0.0001). Fatigue resistance was the highest for group O (1310.8 ± 196.7), and the lowest for group R (905.4 ± 170.51) with a significant difference between groups (*P* < 0.0001). Crown group had the highest percentage (80%) of catastrophic failure, while, overlay group exhibited the lowest (20%).

**Conclusions:**

Direct restoration without cuspal coverage using ribbon fibers with short FRC provided better marginal adaptation than indirect overlays and crowns, but fatigue resistance wasn’t significantly improved. Adhesive ceramic overlays showed the best fatigue performance and the least catastrophic failure rate compared to both direct fiber-reinforced composite and indirect ceramic full coverage restorations.

**Clinical significance:**

Indirect adhesive overlays are a suitable, more conservative restorative option for endodontically treated teeth than full coverage restorations, especially when tooth structure is severely compromised.

## Background

The reconstruction of endodontically treated teeth (ETT) always represents a huge challenge in dentistry due to their increased risk of fracture-related failure; hence, the type of permanent restoration must be carefully chosen [1]. One of the major contributing factors for this high fracture risk is the coronal tooth structure loss due to caries and endodontic procedure. As mentioned in previous literatures, the percentage of reduction in cuspal stiffness is inversely proportional to the remaining tooth structure and directly proportional to the number of missing walls. Where 5–20% decrease in cuspal stiffness was reported for class I occlusal cavity, mesio-occlusal-distal MOD cavity was found to cause elevation of this percentage to 63%. This significant loss of tooth structure can be attributed to the loss of all marginal ridges that reduced the fracture strength by as much as 54%. Thus, Post-obturation restorative materials in endodontically treated premolars with MOD cavities are crucial for protection against future fracture [2]. 

Another contributing reason for failure of ETT is coronal leakage. Marginal integrity of coronal restorations is one of the important factors that help to predict the long-term survival of restorations [3]. For the past few decades, full coverage crowns with intra-radicular posts were used for restoration of ETT. In addition to more weakening of the tooth by post-space preparation and procedural errors such as root perforation, the actual reinforcing efficiency of fiber-reinforced composite posts is a matter of debate [2, 3]. With the emergence of biomimetic concept, a shift towards more conservative treatment planning for ETT became necessary to balance the mechanical, biological, adhesive, functional, and aesthetic parameters without further weakening of the brittle tooth structure. Coronal tissue preservation and avoiding invasive endodontic procedures guarantee biomechanical balance and long-term effectiveness of the restored tooth [4]. Thus, with the recent developments in adhesive protocols, indirect ceramic onlays and overlays became very popular.

The presence of the proximal box can enhance its retention and resistance but optimized preparation will require the removal of healthy dental tissues. Thus, it was recommended that these indirect adhesive designs should be only implemented when a previous restoration existed [4, 5]. 

On the other hand, post-free direct core build-up restorations have gained interest recently [6]. The use of fiber-reinforcement composite restorations (FRCs) had showed promising results in many previous studies [7–9]. The most commonly tested FRCs types are glass combined with ultra-high strength polyethylene fiber ribbon (Ribbond). Owing to minimal invasiveness and simplification of clinical procedures; they have the ability to enhance the fracture strength of restorations of both vital and ETT. Additionally, an improved marginal adaptation, restoration integrity and decreased micro-leakage have been reported [10]. 

Unfortunately, there is still no consensus about the optimal coronal restoration for endodontically treated premolars. Furthermore, comparison between full coverage crowns and reinforced direct composite restorations for restoration of endodontically treated premolars was rarely investigated in literature. Therefore, the aim of this present study is to investigate the effect of three different coronal restorations (direct with polyethylene fibers and glass short-fiber reinforced composite, indirect ceramic adhesive overlay and indirect full coverage ceramic crown) on the marginal behavior and fatigue resistance of endodontically treated teeth. The null hypotheses are that there would be no significant difference in marginal adaptation of all three tested restorative materials and designs. Moreover, restorative material and design would not significantly affect the fatigue resistance of endodontically treated teeth.

## Materials and methods

The materials used for coronal restorations in this study are presented in Table (1). Sample size was calculated using G Power 3.1.9.7., based on the previous study by Hitz et al. [11]. , as reference. According to this study, the accepted sample size was 8 per group, when the mean and standard deviation of group 1 was (90.3 ± 2.6), while the mean and standard deviation of group 2 were (80.4 ± 9) with 1.23 effect size. Total sample size was increased to 10 to compensate for 20% drop out when the power was 80% & type I error probability was 0.05.

**Table 1:** Materials used in coronal restorations and their compositions:

Type	Materials	Main Composition	Manufacturer	Lot no.
Lithium disilicate CAD/CAM glass ceramic blocks	IPS e.max CAD	SiO2 (57–80%), Li2O (11–19%), K2O (0–13%), P2O5 (0–11%), ZrO2 (0–8%), ZnO (0–8%) and coloring oxides (0–12%)	Ivoclar Vivadent, Schaan, Liechtenstein	Z00858
Polyethylene woven fiber	Ribbond	Ultra-high molecular weight polyethylene, Homopolymer H-(CH2-CH2) n-H	Ribbond Inc., Seattle, WA, USA	*=D758U0/$$72C4/16D20210520/F*
Short fiber reinforced resin composite	EverX Posterior	Bis-GMA, Triethyleneglycol dimethacrylate, Silicon dioxide, Barium glass, Glass fiber, Polymethylmethacrylate Trace Photo initiator	GC Europe, Leuven, Belgium	220,830
Nanohybrid resin composite	Filtek™ Z250-xt	Bis-GMA, UDMA, TEGDMA, Bis-EMA, PEGDMA non-agglomerated/non aggregated silica filler, nonagglomerated/ nonaggregated zirconia filler, and aggregated zirconia/ silica cluster filler Filler Load: Wt:78.5%; Vol:63.3%	3 M ESPE Dental Products, MN, USA	NE72611
MDP Universal Bond	Gluma® Bond	10-MDP phosphate monomer, 4 META, dimethacrylate resins, acetone, fillers, initiators, silane	Kulzer GmbH, Hanau, Germany	M010055
Universal Adhesive	Bisco Bond	Bisphenol A Diglycidylmethacrylate (20–50%), Ethanol (30–50%), Methacryloyloxydecyl dihydrogen Phosphate (5–25%), 2-Hydroxyethyl Methacrylate (2–25%), water, Initiators	Bisco Inc, Schaumburg, USA	220,000,389
Ceramic primer	Porcelain primer	3-(Trimethoxysilyl)propyl-2-Methyl-2- PropenoicAcid (1–5%), Ethanol (30–50%), Acetone (30–50%)	Bisco Inc, Schaumburg, USA	2,200,003,403
Dual cure resin luting cement	Duo-link Universal ™	Base: Bisphenol A Diglycidylmethacrylate (10–30%), Urethane Dimethacrylate (10–30%), Ytterbium Oxide-Silica (1–5%), Tetrahydrofurfuryl Methacrylate (1–5%), Trimethylolpropane Trimethacrylate (1–5%), Ytterbium Fluoride (10–20%), Catalyst: Bisphenol A Diglycidylmethacrylate (10–30%), Dibenzoyl Peroxide (< 1%)	Bisco Inc, Schaumburg, USA	2,200,006,991

This study proposal was reviewed and approved by the Institutional Review Board of the Faculty of Oral and Dental Medicine, Ahram Candian University (IRB00012891), Egypt, on 25/6/2023. With approval number IRB00012891 ≠ 71. A total of thirty sound human maxillary first premolars extracted for orthodontic purposes were obtained from the Department of Oral and Maxillofacial Surgery at the Faculty of Dentistry, Ahram Canadian University. Teeth were examined under magnification loups 4.0X and LED headlight (Univet, Italy) for caries, abrasions, cracks or fractures. Teeth with any of these defects were excluded. Chosen teeth were measured for length of 15 + 1.0 mm and similar buccolingual, mesiodistal, and occluso-gingival dimensions using digital caliper with 0.01 mm accuracy (Mitutoyo IP 65, Kawasaki, Japan). Debris were cleaned using ultrasonic scaler, followed by polishing with a rubber cup and pumice. Immersion in 0.1% thymol solution at room temperature was done for 1 day, and finally, teeth were stored at room temperature in deionized distilled water container until the experiment started [4, 5]. 

### Endodontic treatment

Conservative access cavity preparation was done using endo-access round diamond bur (856; Intensiv SA, Switzerland). Following hybrid method, a low-speed contra-angle hand-piece (W&H Dentalwerk Bürmoos GmbH, Austria), was used for a step-down procedure using gates Glidden burs for the first 3 mm. A digital X-ray was used to assess the working length after the insertion of NiTi files. (Mani NiTi K File) A No.40 master apical file was used and a 1 mm steps step-back procedure was performed until file No.60. Sodium hypochlorite (5%, sodium hypochlorite solution) was used after each file to rinse the canal, and paper points are used to dry the canal. The obturation process was done using No.40 gutta-percha points, accessory points (Meta Biomed), and sealer (AD Seal Meta Biomed). After removal of excess gutta-percha, the access cavity was cleaned with alcohol [4]. 

### Sample grouping

30 teeth were randomly divided into three equal experimental groups according to the coronal restoration after endodontic treatment:

Group R: Restored with non-cuspal coverage direct restoration; polyethylene fibers (ribbond), glass fibers reinforced resin composite (ever-x posterior) and a final layer of nano-hybrid composite (*n* = 10).

Group O: Restored with cuspal coverage, indirect lithium disilicate ceramic overlay (*n* = 10).

Group C: Restored with fiber-post, nano-hybrid resin composite restoration and lithium disilicate full coverage ceramic crown (*n* = 10).

For periodontal simulation, each tooth was embedded in molten wax (CAVEX, CAVEX dental, Netherland), 2 mm below the cementoenamel junction (CEJ). After complete wax solidification, teeth placed in a custom-made plastic mold (10 mm radius and 15 mm depth) filled with self-curing acrylic resin (Acrostone; Acrostone dental, Cairo, Egypt) simulating alveolar bone. After initial polymerization, wax remnants were cleaned out from teeth and acrylic, and light-body polyvinyl siloxane impression material (Panasil® Initial contact; Kettenbach GmbH & Co) was injected into the mold. Teeth were re-inserted parallel to its long-axis into the acrylic socket under constant finger pressure to extrude the excess silicon material and create a uniform silicon layer (0.2–0.3 mm) that simulate the periodontal ligament.

For both group R and group O, a thin layer of universal adhesive (All-Bond Universal. BISCO Inc, USA) was applied to the walls of pulp chamber, and light polymerized for 10 s with light curing unit (Elipar™, 3 M ESPE, USA) according to manufacturer instructions. The pulp chamber was then filled with flowable composite (Filtek ™ Flow, 3 M ™ ESPE, USA).

To ensure proper standardization of teeth preparation, biocopy mode on CEREC 3D software (version 4.5, Sirona Dental Systems GmbH, Bensheim, Germany) was used to obtain a base scan of all teeth before preparation. Samples were scanned using Omnicam scanner and base images were obtained and stored on CEREC 4.5 software to allow for superimposition of the biocopy and the post-preparation scans, so, standardized cavity preparation could be verified. Moreover, polyvinyl siloxane silicon index (Elite HD^+^, Zhermack-Germany) was taken for teeth before preparation for verification and for use as occlusal stamp with teeth assigned to direct restoration group (*n* = 10).

All endodontic procedures and teeth preparation steps were performed by the same operator, under magnification loups 4.0X. Moreover, all indirect restorations laboratory fabrication steps were done by the same laboratory technician.

Standardized MOD cavity was prepared for all teeth with mesial and distal gingival cavo-surface margins 1 mm above CEJ. Intra-coronal cavity of 4 mm depth was prepared using short parallel diamond bur (6836KR, Komet, Schaumburg, USA) mounted in high-speed handpiece (Synea WK-900 LT, W&H Dentalwerk Bürmoos GmbH, Austria) under copious amount of water coolant. The buccal and lingual residual wall thickness were adjusted to 1.5 ± 0.2 mm at the height of contour, and the bucco-lingual width of the proximal box was adjusted to 1/3 of the total bucco-lingual tooth dimensions. Internal line-angles were smoothed and all unsupported enamel was removed with subsequent finishing diamond bur (8836KR, Komet, Schaumburg, USA). All prepared cavities were verified for accurate dimensions with digital caliper.

**Fig. 1:**
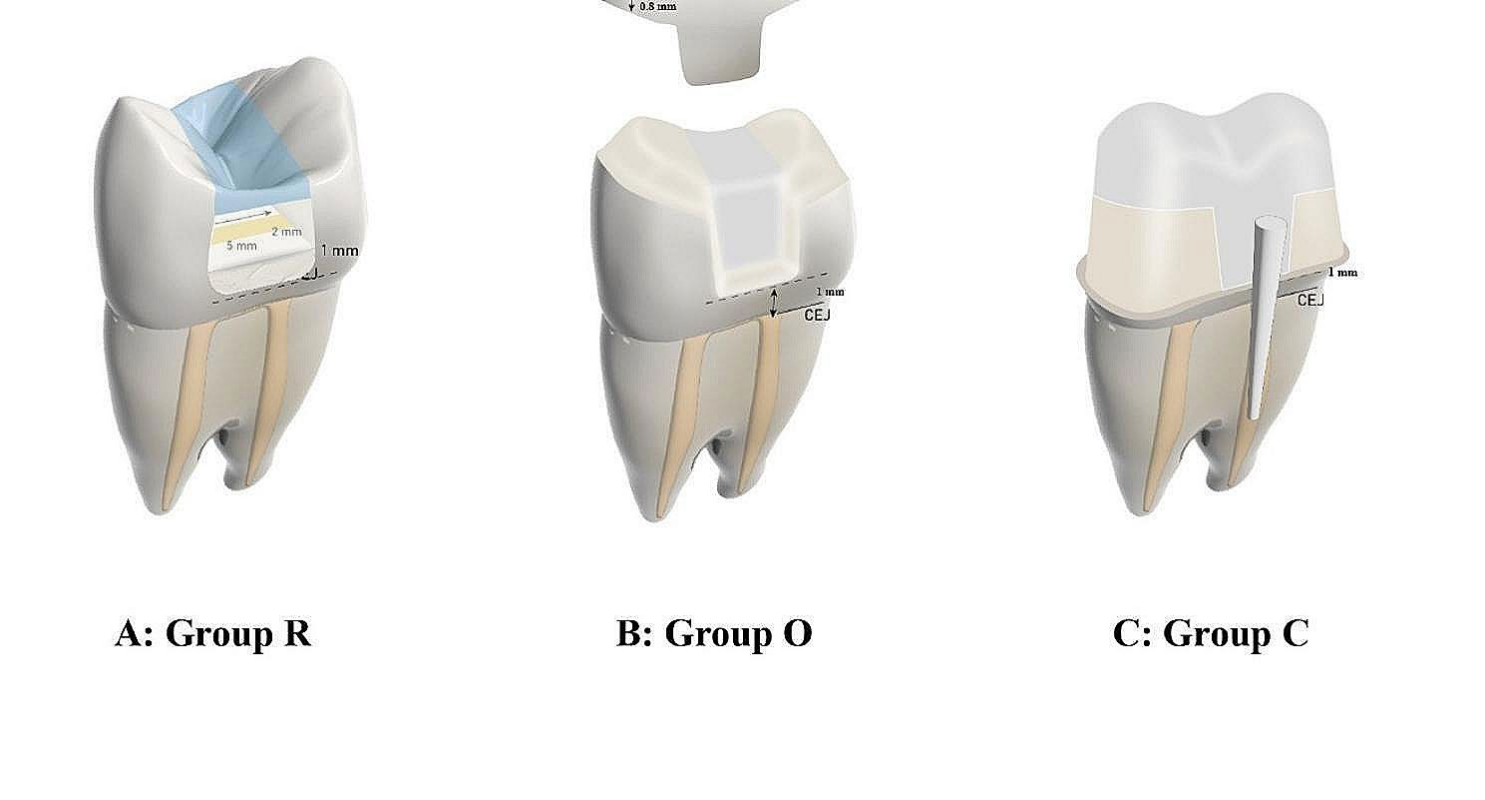
Schematic representation of the three study groups, restored with different approaches: **A**: Ribbond fibers + EverX posterior composite (Group R), **B**: Ceramic overlay (Group O), and **C**: Fiber post + composite build-up + ceramic crown (Group C)

Following this, three different adhesive coronal preparations were carried out for the three study groups (*n* = 10 each), Fig. (1):

### Group R (direct restoration; ribbond + EverX resin composite)

Universal adhesive was applied to the cavity walls and cured, followed by the application of a thin layer of flowable resin composite. A thin strip of Ribbond Ultra (~ 5 mm long, 2 mm wide) was prepared and saturated with MDP containing bonding agent (Gluma® Bond Universal, Kulzer GmbH, Hanau, Germany) and placed in flowable resin in a bucco-lingual direction not touching the enamel margins. After light-curing for 20 s, the remaining cavity space was restored with fiber-reinforced resin composite EverX Posterior (GC Europe, Leuven, Belgium). The last layer with nano-hybrid resin composite (Filtek Z250-xt 3 M ™ ESPE, USA) was applied, then, finishing and polishing were done using discs (Soflex; 3 M ESPE, Germany) [12]. 

### Group O (CAD/CAM- indirect overlay restoration)

Bonding was done to the cavity walls similar to group R, then, the MOD cavity except for the gingival seat (1 mm thickness) was restored with nano-hybrid composite applied in consecutive horizontal layers of maximum 3 mm thickness and light cured for 20 s each as per manufacturer recommendations.

Indirect adhesive overlay preparation was done following morphology driven preparation design (MDPD), with hollow chamfer margin by Veneziani [13]. First, proximal box was adjusted to rounded angles with 8°divergence, 1.5 mm width, 1 mm depth and located 1 mm above the cementoenamel junction using flat-end diamond bur (8845KR, Komet, Schaumburg, USA). Occlusal reduction was done by creating depth orientation grooves using depth-cut bur (DM20, Komet, Schaumburg, USA), followed by OccluShaper diamond bur (KP6370, Komet, Schaumburg, USA) to obtain uniform anatomical occlusal reduction of 1.5 mm, then fine diamond OccluShaper bur (8370, Komet, Schaumburg, USA) was used for occlusal surface finishing. Hollow chamfer margin of 0.8 mm (± 0.2) thickness was prepared using tapered chamfer diamond bur (6856-014, Komet, Schaumburg, USA) and finished with fine grit diamond bur (8856-014, Komet, Schaumburg, USA). All preparation corners were finally rounded with spitz pointed Arkansas white abrasive (307, Komet, Schaumburg, USA) and polisher (9436 M, Komet, Schaumburg, USA).

### Group C: (Fiber post, composite restoration, and lithium disilicate full coverage ceramic crown)

For cylinder-tapered size 2 glass fiber post (FiberKleer™ 4X, Pentron, USA), a 10 mm deep preparation was made in the palatal canal using the corresponding, leaving 5 mm of gutta-percha as apical seal. Universal adhesive was applied to the cleaned and dried post space for 20 s then air dried. Dual cured adhesive resin cement (Duo-link Universal ™, Bisco Inc, Schaumburg, USA), was introduced into the post space with lentulo spiral rotary instrument (Dentsply Sirona, Switzerland), followed by immediate insertion of fiber post under finger pressure for 15 s. After complete removal of excess cement, light polymerization was done for 40 s. Cavities were bonded and restored with nano-hybrid composite as in group O [14]. 

For full coverage crown preparation, a standardized planner occlusal reduction of 2 mm was achieved. Axial reduction with heavy chamfer margin 1 ± 0.5 mm above cementoenamel junction was done with tapered chamfer diamond bur and finished with similar size fine grit diamond bur. All preparation corners were finally rounded with spitz pointed Arkansas white abrasive [15]. Preparations for all indirect adhesive restorations in both Group C and O were checked by digital caliper and digitally verified using CEREC software via PrepCheck feature (version 4.5, Sirona Dental Systems GmbH, Bensheim, Germany).

### Digital workflow, fabrication of indirect restorations and final cementation

Omnicam intraoral scanner (Dentsply-Sirona, Bensheim, Germany) was used for scanning of the prepared teeth from the two indirect restorations groups (Group O and Group C). Restorations were designed using CEREC 3D software (version 4.5, Dentsply-Sirona, Bensheim, Germany). To standardize the design with similar occlusal morphology and average thickness of 1.5 mm at the cusp tip, and 1 mm fissure thickness, biogeneric reference tool in CEREC software was utilized. It allows designing all restorations from a previously stored dentoform maxillary first premolar scan. Only position tool was used for restoration adjustment to avoid any alteration of restoration’s original proposal. Cement space of 80 μm to allow precise restoration seating without marginal discrepancy [16, 17]. 

All restorations were milled from lithium disilicate blocks (IPS e.max CAD, Ivoclar Vivadent, Schaan, Liechtenstein) using MCXL 4-axis wet milling and grinding machine (Dentsply Sirona, Bensheim, Germany). After checking of thickness with digital caliper, crystallization and glazing were done in ceramic furnace Programat P310 (Ivoclar Vivadent Inc., New York, USA) following the recommended manufacturer parameters.

Adhesive cementation of indirect restorations was done following the manufacturer recommendations for IPS e.max CAD. The intaglio surface of each restoration was etched with buffered hydrofluoric acid gel 9.5% (Bisco porcelain etchant, Bisco, USA) for 20 s followed by rinsing for 20 s and another 20 s of air drying. A thin layer of silane primer (Porcelain primer, Bisco, USA) was applied to the etched surface and left for 30 s then air dried. Prepared teeth were etched with for 30 s with 37% phosphoric acid gel (Etch-37, w/BAC, BISCO Inc, USA), rinsed and air died. Followed by application of bonding agent and light polymerization for 10 s. All restorations were adhesively cemented to their corresponding teeth samples using dual-cure luting resin cement (Duo-link Universal ™, Bisco Inc, Schaumburg, USA) and a custom-made loading device was used to apply a vertical load of 1 kg. Initial curing for 3 s was done followed by removal of excess cement and 40 s curing on each surface. Finally, all samples were incubated in distilled water for 24 h before thermal cycling.

### Marginal gap assessment

**Fig. 2:**
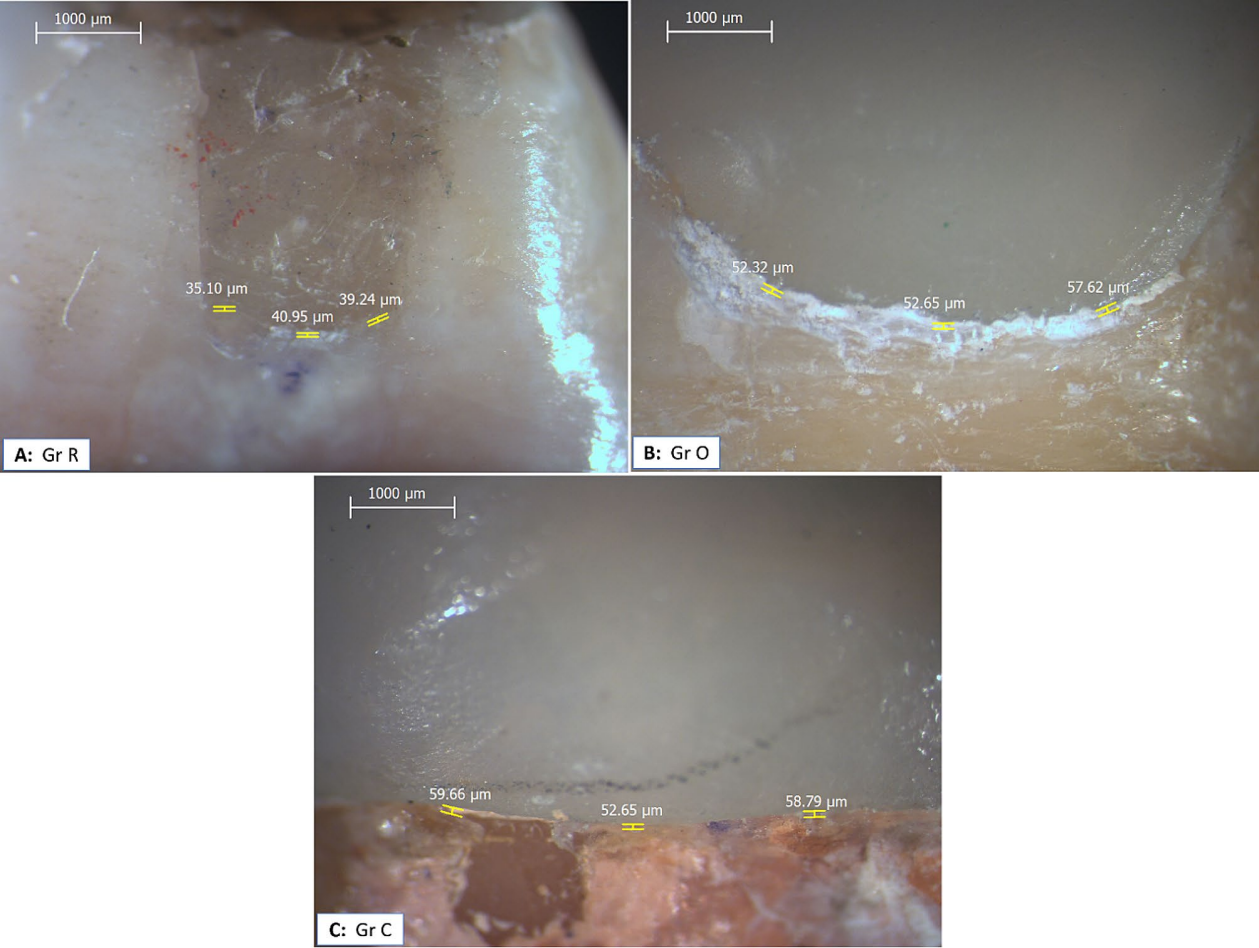
Stereomicroscopic images showing marginal gap measurement in different groups before thermal cycling. **A**: Group R, **B**: Group O, and **C**: Group C

Marginal gap assessment before thermal cycling was done with direct viewing method using stereomicroscope (Leica MZ6, Leica Microsystems, ETH Zurich, Switzerland) with 30x magnification [18–21]. High definition digital camera was utilized for capturing images (Leica MC190 HD, Leica Microsystems, Switzerland). Four images were obtained for each sample (one for each axial surface). Images were uploaded to the image analysis software (Image Pro-plus V.6) for marginal gap measurement. Vertical marginal gap along each restoration surface was measured at 3 equal distance landmarks, Fig. (2). Three measurements were taken at each location [20]. All data were collected and tabulated to be used later.

### Thermal cycling

All teeth were subjected to 5000 thermal cycles in masticatory simulator (Robota automated thermal cycle; BILGE, Turkey), equivalent to 6 months. In order to mimic the temperature changes occurring in the oral cavity, dwell periods in each water bath were set at 25 s with 10 s lag time. The minimum temperature was 5 °C. The maximum temperature was 55 °C [20]. 

**Fig. 3:**
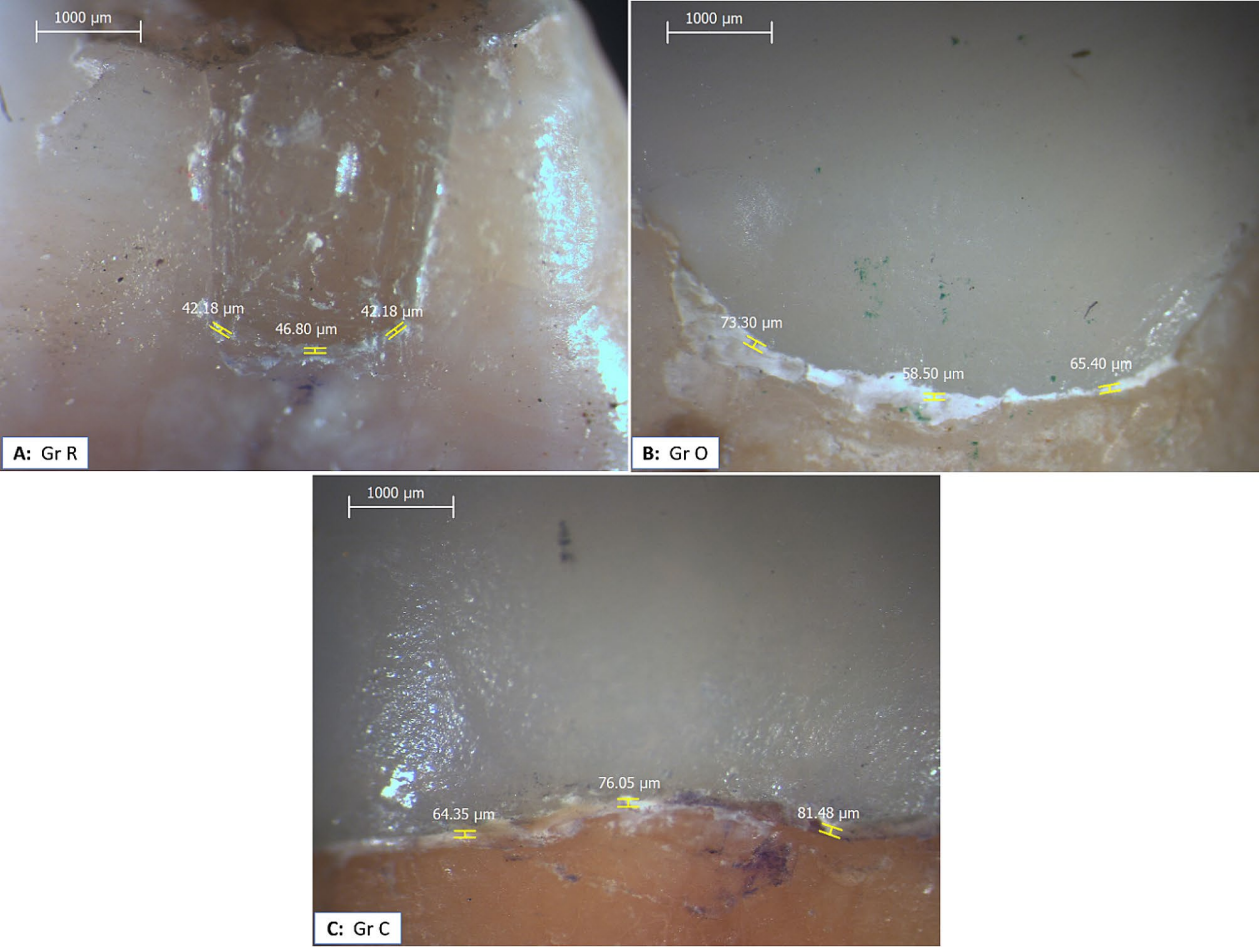
Stereomicroscopic images showing marginal gap measurement in different groups after thermal cycling. **A**: Group R, **B**: Group O, and **C**: Group C

Marginal gap was assessed for all samples after thermal aging with the same method and at the same pre-aging sites, Fig. (3)

### Fatigue resistance test

Resistance to fracture under cyclic loading for all three study groups was done with stepwise stress fatigue method, using a computer-controlled testing machine (Model 3345; Instron Industrial Products, Norwood, USA) with 5kN load cell. Each sample was secured through the acrylic block to the lower part of the testing machine. A metallic stylus with 5 mm diameter of a spherical head and attached to the movable upper part was used to apply compressive load in a vertical direction to the occlusal surface of each sample. After simultaneous contact of the buccal and palatal cusps of the tooth with the spherical head was verified, 0.2 mm thick tin foil was inserted between the stylus head and the tooth to decrease the stress concentration and to mimic the presence of food. The cyclic loads to a maximum of 5000 cycles were applied to each sample at a crosshead speed of 0.5 mm/ minute and 1.6 Hz frequency. Each sample was subjected to a prescribed number of cycles at each of a sequence of increasing stress levels, until failure occurred. At the start of stepwise stress test, a load level below the expected fatigue failure for the material was selected (40% of the average static fracture force). The maximum number of cycles at each step load was set in 1000 cycles. If the specimen survived 1000 cycles, the stress level was raised by a constant load increment (100 N) in the same specimen. 200 N were used as initial load, followed by successive steps of 100 N each [16]. The load and number of cycles required for failure in each sample were recorded using computer software (Bluehill Lite; Instron Instruments). Calculation of the maximum fatigue load (L_E_) supported by each specimen was done according to the equation represented by Nicholas T [22]:

Where: L_0_ = Previous maximum fatigue load which did not cause failure, ∆ L = Load step increase, N_fail_ = Number of cycles till failure (L_0_ + L_E_), N_life_ = Defined cyclic fatigue life (5000 cycles).

The number of intact samples and samples that failed were counted with each load step to calculate the survival probability (%) for each group.

For failure mode analysis, representative samples were examined with stereomicroscope at 10x magnification. Failure modes were categorized into three types according to their type and location: Type I (repairable fracture involving restoration only), Type II (repairable fracture involving restoration and tooth above CEJ), and Type III (Catastrophic, non-repairable fracture involving tooth and restoration below CEJ) [14]. 

### Statistical analysis

Statistical analysis was performed with SPSS 16 ® (Statistical Package for Scientific Studies), Graph pad prism & windows excel. Data exploration was done with Shapiro-Wilk test and Kolmogorov-Smirnov test for normality which revealed that all data originated from normal data. For marginal adaptation, comparison between the three study groups was performed by One Way ANOVA test followed by Tukey`s Post Hoc test for multiple comparison. Comparison between before and after thermal cycling was performed by using Paired t test. For fatigue resistance, comparison of the fatigue failure load between the study groups was done by One-Way ANOVA test, followed by Tukey`s Post Hoc test for pairwise comparison. Life table survival analysis was performed to assess the survival probability (%) of samples in each group at each time interval (represented by each load step). Failure mode analysis (%) comparison was done by Chi-square test. All results were presented as frequency and percentages and all comparisons were performed by using the Chi-square test. The significance level of *p* < 0.05 was set for all tests.

## Results

### Marginal gap evaluation

#### Effect of restoration material and design

**Fig. 4:**
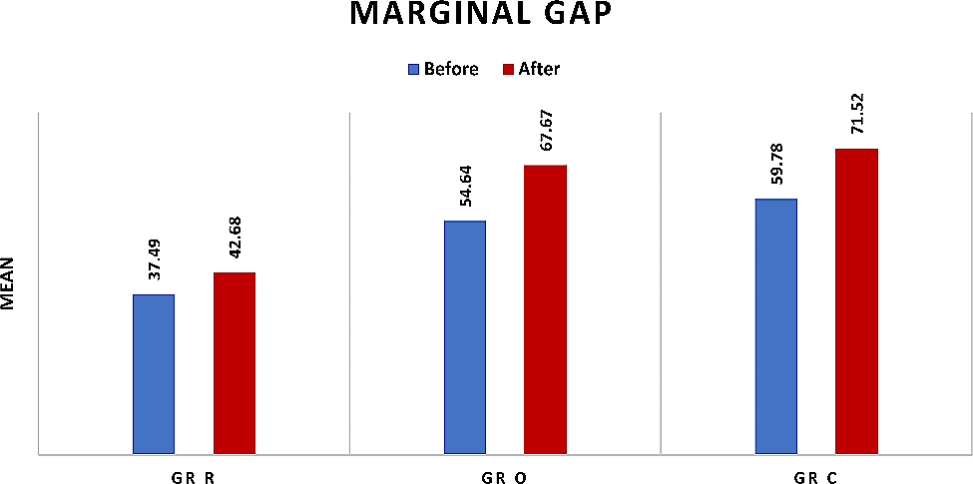
Bar chart showing Marginal gap in Group R, group O, and Group C before and after thermal cycling

Comparison between all groups revealed high statistically significant difference as *P* < 0.0001in both before and after thermal cycling. Multiple comparisons revealed that Group R was significantly the lowest (37.49 ± 5.05) and (42.68 ± 2.38), Group O (54.64 ± 4.79) and (67.67 ± 8.64), while Group C (59.78 ± 5.67) and (71.52 ± 5.18) were significantly the highest with insignificant difference between them regarding before and after thermal cycling respectively, as presented in Table (2), and Fig. (4)

#### Effect of thermocycling

Comparison between before and after thermocycling revealed significant increase in marginal gap in all groups as *P* = 0.01,0.001 and 0.0009 regarding Group R, Group O, and Group C respectively, as presented in Table (2), and Fig. (4)

**Table 2:** Marginal gap in Group R, group O, and Group C before and after thermal cycling:

	Before thermocycling		After thermocycling		Paired differences				
					MD	SEM	95% CI		***P*** **value**
	M	SD	M	SD			L	U	
Gr R	37.49 b	5.05	42.68 b	2.38	4.91	4.99	1.35	8.49	0.01*
Gr O	54.64 a	4.79	67.67 a	5.90	12.8	8.64	6.61	18.98	0.001*
Gr C	59.78 a	5.67	71.52 a	5.18	7.72	2.44	6.33	17.4	0.0009*
*P* value	< 0.0001*		< 0.0001*						

M: mean SD: standard deviation MD: mean difference SEM: standard error mean CI: confidence interval L: lower arm U: upper arm *Significant difference as *P* < 0.05 ** high significant difference as *P* < 0.001.

### Fatigue failure load (N)

**Fig. 5:**
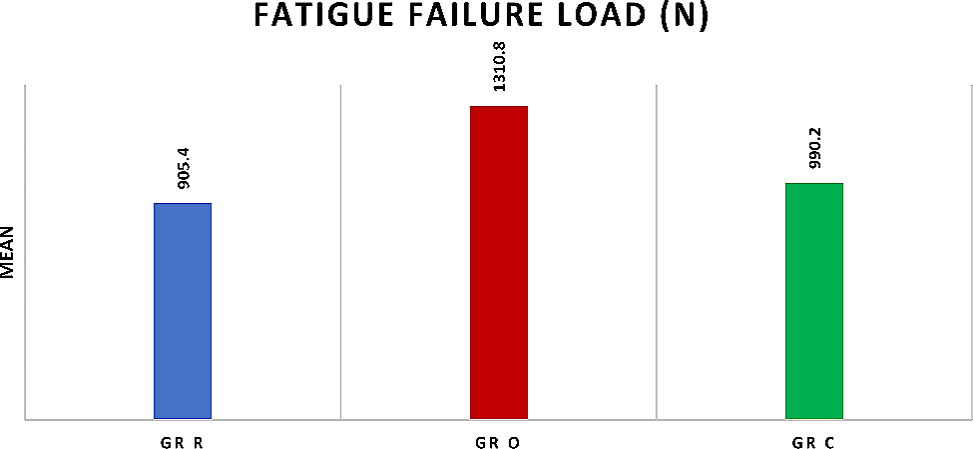
Bar chart showing Fatigue failure load (N) in Group R, group O, and Group C

Comparison between different groups revealed high statistically significant difference between them as *P* < 0.0001, multiple comparisons revealed that Group O (1310.8 ± 196.7) was significantly the highest, while Group R (905.4 ± 170.51) and group C (990.2 ± 160.47) were significantly the lowest with insignificant difference between them, as presented in Table (3), and Fig. (5)

**Table 3:** Fatigue failure load (N) in Group R, group O, and Group C:

Fatigue Failure Load (*N*)	Min	Max	M	SD	*P* value
Gr R	708.7	1102.1	905.4 ^**a**^	170.51	< 0.0001**
Gr O	910	1702.5	1310.8 ^**b**^	196.7	
Gr C	711.1	1302.5	990.2 ^**a**^	160.47	

M: mean SD: standard deviation ** high significant difference as *P* < 0.001.

Means with the same superscript letters were insignificantly different as *P* > 0.05.

Means with different superscript letters were significantly different as *P* < 0.05.

**Fig. 6:**
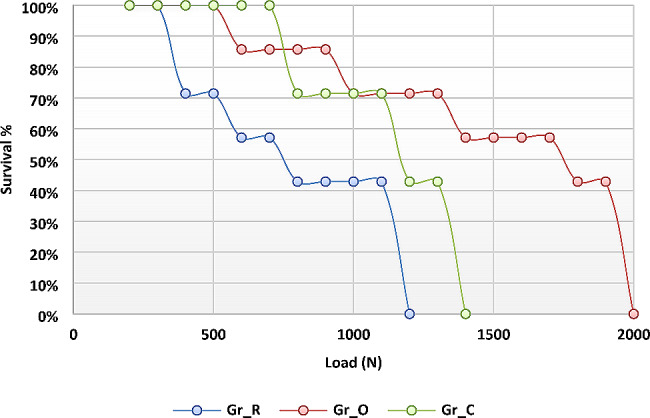
Life table survival distribution by the different coronal restorations at each load step

Life table survival analysis is presented in Fig. (6), shows the survival distribution by the three groups at each load step. Survival performance was the highest for group O followed by group C, while the lowest survival performance was recorded with group R.

### Failure mode analysis

**Fig. 7:**
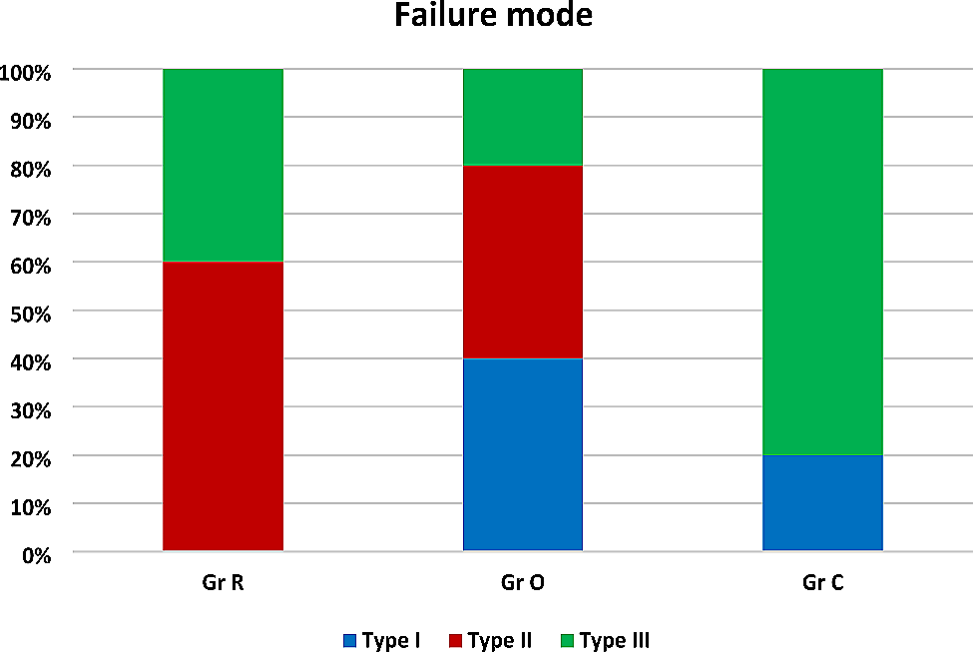
Stacked bar chart representing distribution of all failure modes in different groups

**Fig. 8:**
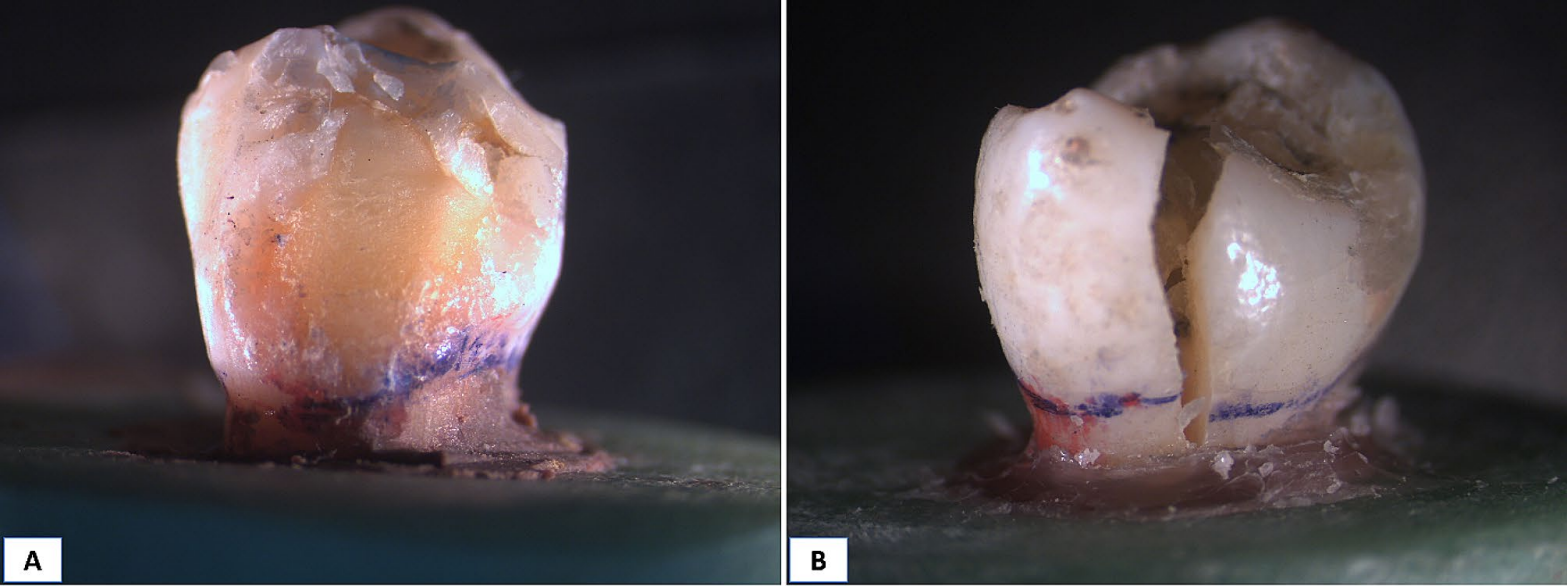
Stereomicroscopic images showing failure modes in Group R. **A**: Repairable type II, and **B**: Catastrophic type III.

**Fig. 9:**
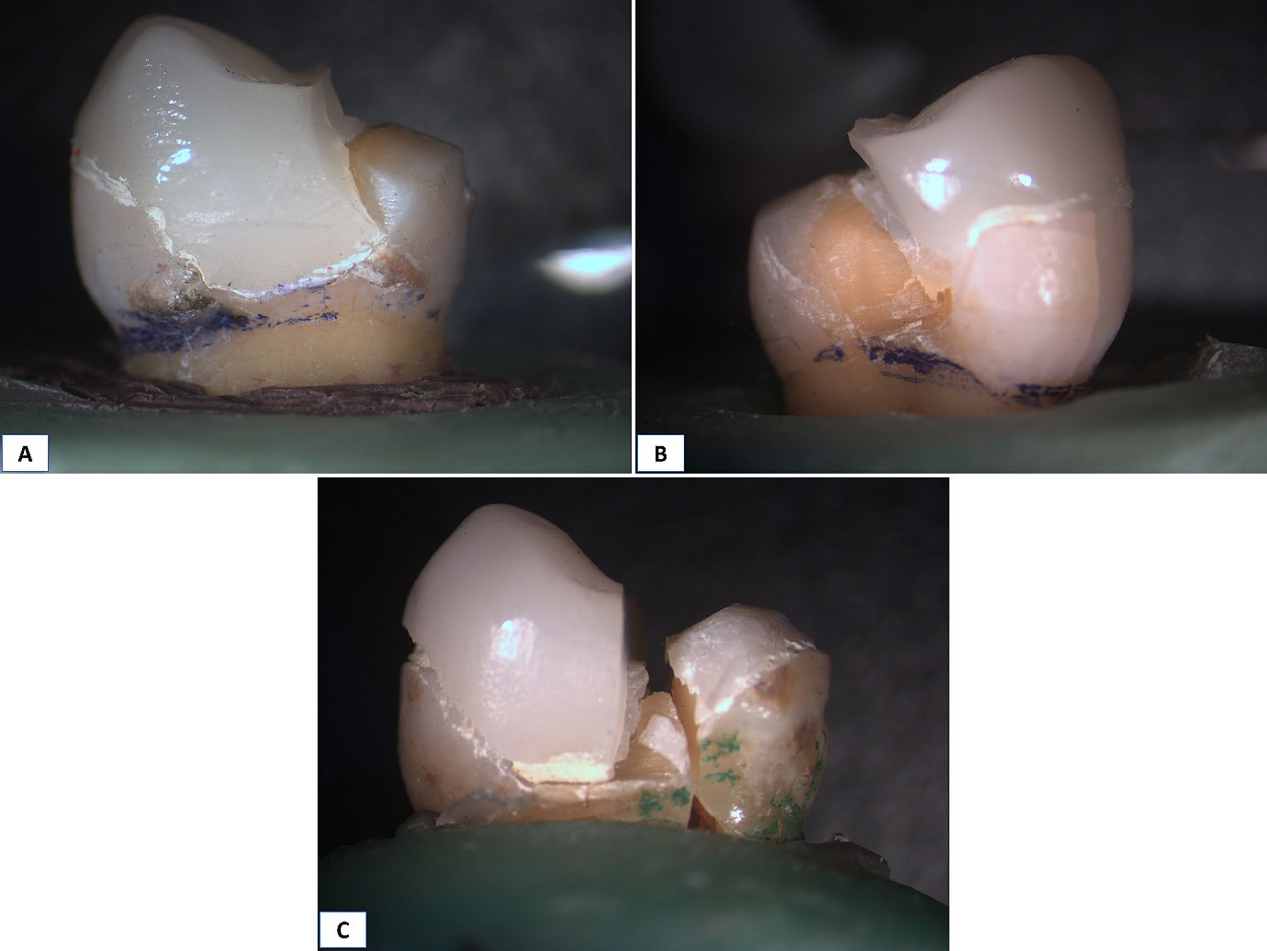
Stereomicroscopic images showing failure modes in Group O. **A**: Repairable type I, **B**: Repairable type II, and **C**: Catastrophic type III.

**Fig. 10:**
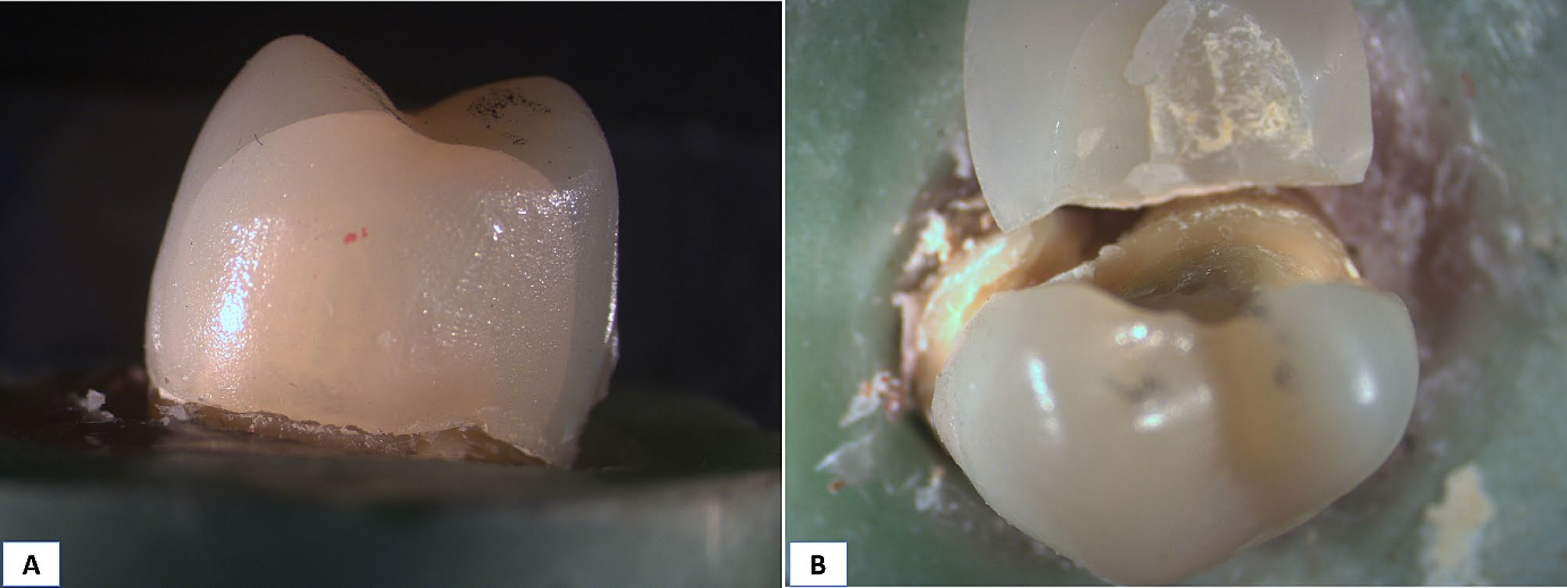
Stereomicroscopic images showing failure modes in Group C. **A**: Repairable type I, and **B**: Catastrophic type III.

Comparison between the three different groups revealed high statistically significant difference between them as *P* < 0.0001, multiple comparisons revealed that Group C exhibited the highest percentage of non-repairable fractures (Type III) (80%), while group R recorded (40%) and group O was significantly the lowest (20%) with a significant difference between them, as presented in Table (4), Fig. (7) and stereomicroscopic images Figs. (8, 9 and 10)

**Table 4:** Frequency and percentages of different failure modes in all groups and comparison between them using Chi square test:

Failure mode	Type I		Type II		Type III		P value
	N	%	N	%	N	%	
Gr R	0	0	6	60	4	40	0.009**
Gr O	4	40	4	40	2	20	
Gr C	2	20	0	0	8	80	

N: frequency %: percentages ** Highly significant difference as *P* < 0.001.

## Discussion

The primary causes of failure of endodontically treated teeth are coronal leakage and fracture of compromised remaining tooth structure [2, 5]. In addition, cuspal coverage restorations such as crowns and overlays were frequently recommended over non cuspal coverage indirect inlays and conventional direct composite restorations due to high rate of non-repairable failure of the later [5, 12, 14]. Hence, this study aimed to investigate the marginal quality and fatigue fracture behavior of endodontically treated premolars restored with two indirect cuspal coverage restorations with a direct reinforced intra-coronal restoration.

Marginal adaptation results in this study indicated significant difference between the three tested groups. Thus, the first null hypothesis regarding the effect of restorative material and design on marginal adaptation was rejected. In this study, meticulous measures were taken to simulate clinical sitting as much as possible, starting from natural teeth selection, preparation standardization followed by verification both by digital caliper as well as 3D verification on CAD/CAM software. In addition, high accuracy intraoral scanner was used for final impression of all indirect restoration to avoid possible discrepancies from conventional impression technique [19]. Direct viewing with stereomicroscope was used for assessment of vertical marginal gap in this study, which is considered faster, non-destructive as well as more affordable and requires fewer procedural steps in comparison to the indirect technique [18, 20]. Vertical marginal gap for all study samples was assessed twice; before and after thermal aging, for a close imitation of clinical conditions. All steps of teeth preparation, restoration fabrication, cementation, and measurements for marginal gap were done by the same operator to avoid any procedural errors as much as possible.

The results of this study revealed that vertical marginal gap values recorded for all study groups before and after thermal cycling were within the clinically accepted range < 120 μm that was previously set McLean and von Fraunhofer [23]. 

Our results showed that direct restoration without cuspal coverage using ribbon fibers with short FRC resin matrix yielded a significantly less marginal gap values (Group R) in comparison to indirect ceramic overlay (Group O) and full coverage crown (Group C), both before and after thermal aging. This could be explained by the ability of polyethylene fibers to decrease the volumetric shrinkage of composite, hence, prevent the separation of composite material from the gingival margin of MOD cavity during polymerization [6]. These results were in agreement with Althaqafi k [24]. , who reported an increased marginal gap and subsequent microleakage with CAD/CAM indirect onlays fabricated from two different machinable composite blocks in comparison to direct everX posterior composite onlays. These findings were explained by the presence of resin cement layer in indirect restoration which contributed to the presence of additional space as well as voids at the tooth restoration interface.

CAD/CAM ceramic material is another contributing factor to the marginal adaptation results. Lithium disilicate ceramic material (IPS e.max CAD), was used in this study for fabrication of overlays and full coverage crowns. Lithium disilicate is supplied in a pre-crystallized metasilicate state which is easier for milling, then, the milled restoration undergoes final crystallization in porcelain furnace to reach its maximum flexural strength. These processing steps may result in some dimensional changes in the final restoration, which may lead to a slight increase in the vertical marginal gap, but still within the clinically acceptable threshold [25]. 

Contrary to our results, Hitz et al. [11]. , found no significant difference in vertical marginal gap between FRCs and inlay restoration before thermal cycling. This could be explained by the difference in glass fiber and resin composite from those used in our study. In addition, they used leucite-reinforced glass ceramic (ProCAD) for inlay construction, which is more prone to chipping than lithium disilicate ceramic used in our study, which may explain the increased marginal gap mean values in both groups from our study.

Among other factors that may influence the marginal adaptation of indirect restorations are preparation design and margin configuration. In this study, indirect overlays were prepared following MDPD, which allows for the development of smooth occlusal surface and non-retentive preparation with fewer internal angles. These preparation features allow for easier digital scanning, designing and final milling of the restoration with high details reproduction, subsequently improving the margin quality in comparison to retentive full coverage preparation [13]. Furthermore, the presence of hollow chamfer margin in overlay preparation increases the surface area, while facilitating homogenous transition between the internal line angles, proximal box, and finish line area. This was reported to allow for better restoration seating with thin cement gap and improve the overall marginal adaptation, which may have contributed to lower marginal gap values in overlay group in comparison to full coverage crown group with heavy chamfer margin design [26, 27]. Dere et al. [28]. , explained good margin quality of overlay restorations by the reduction of the cavity configuration factor (C-factor) due to cusp shortening. When combined with the presence of thin cement thickness, smaller C-factor resulted in lower polymerization shrinkage at the margin area.

Thermal cycling was reported in many studies to have a detrimental effect on the marginal adaptation of both composite and ceramic restorations [18]. The results of our study revealed significant increase in marginal gap mean values in all groups following thermocycling. Group R showing the lowest gap values while group C showed the highest gap mean values. Previous studies attributed the margin deterioration after aging in fiber ribbon group to the vertical shrinkage of composite due to thermal fluctuation despite the presence of horizontally aligned fibers that keep horizontal shrinkage to minimum [6, 24, 29]. 

On the other hand, higher gap values in both indirect restoration groups after thermal cycling with the highest being in group C, could be attributed to the cement degradation by aging. This usually contributes to higher marginal gap which is further amplified by the difference in thermal expansion coefficient of resin cement from that of restoration ceramic material [26]. Similar results were reported by other studies for overlays [24, 30], and ceramic full coverage restorations following artificial aging [31]. However, our results disagree with those reported by Angerame et al. [17]. , who found no difference in marginal adaptation of ceramic overlays before and after aging. Furthermore, a non-significant marginal deterioration in full coverage crowns after thermomechanical aging was reported by Stappert et al. [32]. , which is also in contradiction with our results.

The second part of this study was testing the fatigue performance of the three restorative approaches. Fatigue resistance results in this study revealed high statistically significant difference between the study groups, hence, the second null hypothesis was also rejected.

The selection of maxillary premolars with MOD cavity design in this study was done in order to simulate the worst-case scenario in posterior teeth after root canal therapy as much as possible. The combination of unfavorable anatomy in terms of crown volume and crown/root proportion with the complex lateral forces amplified by their dental arch location, were frequently reported in literature to increase premolars susceptibility to cusp deflection and fracture than molars [14]. Periodontal simulation was done in all study samples as it can affect the load-bearing properties of the tooth. The maximum normal masticatory force range in maxillary premolar area is 222–445 N. While, in cases where parafunctional habits exist, masticatory forces could reach 800 N [33]. The mean fatigue failure load for the three groups in this study (905.4 N, 1310 N, and 990.2 N) exceeded both normal and parafunctional masticatory loads, hence, a good clinical performance of all the tested restorations can be predicted.

The results of this study revealed that overlay group had the highest mean value of fracture resistance and the highest survival percentage. This could be attributed to the fact that fewer axial walls are present in the final overlay preparation in comparison to full coverage crown preparation. This was in accordance with Huang et al. [33]. , who explained the inversed proportion between the number of restored axial walls and the fracture resistance. Moreover, the conservation of remaining sound tooth structure with MDPD of overlays, increased the surface area for adhesion and improved the distribution of occlusal load which improved the overall fracture resistance of the tooth-restoration complex as was also reported by Abbas & Abulameer [5]. and Sirous et al. [27]

Conversely, Alberto-Jurado et al. [34]. , reported higher load values in complete coverage than the overlay restorations. They explained this by the theory of “plates and shells”. This theory suggested that the entire restoration is responsible for the load-bearing ability not only the top or bottom parts. Therefore, the presence of larger surface in full coverage will result in better stress distribution and higher fracture resistance than any partial coverage restoration. This variation from our results could be attributed to using different substrates. In the previous study, epoxy resin dies were used which may be responsible for different fracture resistance results.

A non-significant difference in post-fatigue fracture resistance was found between group R and group C. The high fracture resistance in the conservative direct restoration group could also be attributed to cusp strengthening by the presence of the horizontal fibers, which decrease cusp deflection and improve the stress distribution. Karzoun et al. [35]. , reported similar results which they explained by the low elastic modulus of the fibers which mimics that of natural dentine. On the other hand, Frankenberger et al. [36]. , reported significantly lower fracture resistance for direct FRCs in comparison to CAD/CAM overlays and full coverage crowns. They also reported a non-significant difference between overlays and full coverage crowns, but suggested that conservative preparation for partial coverage is better utilized in order to preserve the remaining tooth structure. This difference in results may be due to the difference in the type of fiber-reinforcement and the application technique. In our study, ribbond fibers were used followed by everX posterior composite, while they used everX posterior only.

It’s worth mentioning that previous studies solely compared conventional composite with short fiber composite or ribbon fibers addition to conventional composite [10, 37, 38]. Mangoush et al. [10]. , reported an improved fracture resistance, marginal adaptation and decreased microleakage in both short fibers composite and ribbon reinforced conventional composite in comparison to conventional composite alone.

On the other hand, no previous study investigated the effect of adding ribbon reinforcement to short fiber composite. Our study aimed to investigate the claims that combining both materials as a post-endodontic direct coronal restoration would resemble indirect cuspal coverage restorations in survival and marginal seal, while preserving sound tooth structure [39]. Fiber pre-impregnated ribbon (Ribbond) is subjected to cold gas plasma treatment in order to enhance their chemical bond to the resin composite restoration. Those fibers provide multi-directional reinforcement and absorb the applied load, thereby, splinting the weakened tooth internally. Moreover, the short FRC (EverX posterior) has the ability to simulate the shock-absorbing properties of natural dentine [38]. Short fiber (everX posterior) composites act by “isotropic reinforcement” effect. They provide three-directional reinforcement owed to the content of randomly oriented short glass fibers in addition to the inorganic fillers, which are able to deflect and abort the crack propagation in composite restoration [10]. However, the survival rate of group R was still lower than other two indirect restoration groups where cusp coverage was provided. Which proves that non-cuspal coverage restorations do not offer complete protection to EET under fatigue loading, hence, long term survival is questionable as was previously reported by Fráter et al. [14]

Failure mode analysis revealed that indirect adhesive overlays had the least incidence of catastrophic non-repairable fractures (20%), in comparison to group R (40%) and group C (80%). This could be attributed to the concentration of the tensile stresses in the brittle ceramic restoration, which caused cohesive failure of the restoration without fracture of the tooth structure (Type I) [33]. The preparation design of overlay which only involves part of the tooth structure while leaving a large intact part above the CEJ could also be another contributing factor to low catastrophic failure percentage in overlay group. 40% of overlays were fractured with the underlying tooth structure above CEJ (Type II), which represents a much favorable outcome in an indirect ceramic restoration than the catastrophic pattern (Type III) that was predominant in full coverage ceramic crown group. These results were in agreement with Haung et al. [33]. However, fracture analysis results reported by Frankenberger et al. [36]. , along with Abbas and Abulameer [5], disagree with our results, which could be attributed to the difference in the restoration materials and substrates used.

### Limitations

Among the limitations of this study is the fatigue testing set-up. Load was applied in vertical direction to the center of the occlusal surface of the tooth, while, masticatory loading is more complex than that. In addition, only maxillary premolars were investigated in this study. As the search for the optimum restoration for endodontically treated teeth is continuous, further studies are needed to investigate the behavior of similar treatments for teeth in other positions in the dental arches. Furthermore, additional clinical studies are needed to be able to directly correlate the results of this study to the actual clinical conditions.

## Conclusion

Based on the results gained from this study, we can conclude that:

1. Direct restoration without cuspal coverage using ribbon fibers with short FRC resin matrix provided better marginal adaptation than both indirect ceramic overlays and full coverage crowns.
2. Marginal adaptation of all the tested materials was within the clinically accepted range in both before and after artificial aging.
3. Indirect adhesive ceramic overlays showed the best fatigue performance, highest survival rate and the least catastrophic failure compared to both direct fiber-reinforced composite and indirect ceramic full coverage restorations. It can be considered a suitable, more conservative restorative option for endodontically treated teeth than full coverage restorations, especially, when tooth structure is severely compromised.
4. Post-fatigue fracture performance of all the tested materials exceeded both normal and parafunctional masticatory forces in maxillary premolar region.

## Data Availability

The datasets used and/or analyzed during the current study are available from the corresponding author on reasonable request.

## Abbreviations

EET: Endodontically treated teeth
MOD: Mesio-occlusal-distal
FRCs: Fiber-reinforcement composite restorations
CEJ: Cementoenamel junction
MDPD: Morphology driven preparation design
